# The bioactivities of sclareol: A mini review

**DOI:** 10.3389/fphar.2022.1014105

**Published:** 2022-10-03

**Authors:** Jianbo Zhou, Xiaofang Xie, Hailin Tang, Cheng Peng, Fu Peng

**Affiliations:** ^1^ Key Laboratory of Drug-Targeting and Drug Delivery System of the Education Ministry and Sichuan Province, Engineering Laboratory for Plant-Sourced Drug and Sichuan Research Center for Drug Precision Industrial Technology, West China School of Pharmacy, Sichuan University, Chengdu, China; ^2^ State Key Laboratory of Southwestern Chinese Medicine Resources, Chengdu University of Traditional Chinese Medicine, Chengdu, China; ^3^ Department of Breast Oncology, Sun Yat-sen University Cancer Center, Guangzhou, China

**Keywords:** sclareol, bioactivities, cancer, inflammation, delivery

## Abstract

Sclareol, a diterpene alcohol isolated from the herbal and flavor plant clary sage (Salvia sclarea L.), is far-famed as the predominant ingredient in the refined oil of Salvia sclarea (L.). The empirical medicine of *Salvia sclarea* L. focused on various diseases, such as arthritis, oral inflammation, digestive system diseases, whereas the sclareol possessed more extensive and characteristic bioactivities, including anti-tumor, anti-inflammation and anti-pathogenic microbes, even anti-diabetes and hypertension. However, there is a deficiency of literature to integrate and illuminate the pharmacological attributes of sclareol based on well-documented investigations. Interestingly, sclareol has been recently considered as the potential candidate against COVID-19 and Parkinson’s disease. Accordingly, the bioactive attributes of sclareol in cancer, inflammation, even pharmacochemistry and delivery systems are reviewed for comprehensively dissecting its potential application in medicine.

## Introduction

*Salvia sclarea* L (SSL, S. sclarea), known as clary sage, plays pivotal role in herb medicine and essential oil industry (Chalvin et al., 2021a). Notably, its essential oil has been investigated for various bioactivities including anti-oxidant, anti-bacterial, anti-fungal, anti-inflammatory, anti-diabetic, and so on (Öğütçü et al., 2008; Yuce et al., 2014; Durgha et al., 2016; Raafat and Habib, 2018). According to the early literatures, S. sclarea was widely applied in the empirical medicine for treatment of various diseases, such as arthritis, oral inflammation, digestive system diseases and dysmenorrhea (Peana and Moretti, 2002; Kostić et al., 2017). The remediation potential of SSL in metal polluted soils has been revealed, especially under Cadmium stress and zinc tolerance (Chand et al., 2015; Dobrikova A. et al., 2021; Dobrikova A. G. et al., 2021).

Sclareol (SCL, Labd-14-ene-8, 13-diol), a diterpene alcohol enriching in capitate oil glands of calyxs, was mainly isolated from inflorescences of *Salvia sclarea* L (Balinova-Tsvetkova and Tsankova, 1992; Schmiderer et al., 2008; Caissard et al., 2012). It also was indispensable raw materials in the synthesis of Ambrox (ambroxide) (Günnewich et al., 2013). SCL, accounting for about 11.5–15.7% in the essential oil, was produced *via* the two steps enzymatic reaction of Diterpene Synthase (diTPs) and a class II diTPs to substrate Geranylgeranyl Diphosphate (GGPP) and released from chloroplast (Farka et al., 2005; Caniard et al., 2012; Günnewich et al., 2013; Durgha et al., 2016). The terpenoid compositions content including SCL in S. sclarea were affected by geography, climate, temperature, carbon dioxide, nitrogen, plant lines, etc (Yadav et al., 2010; Kaur et al., 2015; Kumar et al., 2017; Tuttolomondo et al., 2021). SCL also existed in several other plant species, comprising *Cistus creticus* (Cistaceae), *Nicotiana glutinosa* (Solanaceae) and *Cleome spinosa* (Brassicaceae) (Caniard et al., 2012). Recently, SCL was identified one of the components of aromatic extraction products (6.9%) obtained from *Nicotiana glutinosa* L (Popova et al., 2019). As a natural flavor, SCL is widely used in cosmetics and food industry. Salvia sclarea L is widely planted for the extraction of SCL based on commercial purpose for its high content of SCL. SCL performed antiphotoaging efficacy *in vitro,* and exhibited wrinkle improvement effect in clinical test (0.02% sclareol-containing cream). Furthermore, SCL inhibited ultraviolet-B inducing MMPs expression and prevented collagen degradation by down-regulating the protein expression of AP-1 transcription factors (Park et al., 2016).

There are three synthesis routes of Ambrox from sclareol, in which the classical commercial route including three reactions and two intermediates sclareolide and ambradiol (Yang et al., 2016). However, the one-pot synthesis was viewed to be convenient and environmentally friendly. Another strategy using strains, containing *Cryptococcus albidus* and *Hyphozyma roseonigra*, to transform sclareol to sclareol glycol, and then the latter was converted to Ambrox using chemical conversion (Wang et al., 2019). Here, we first summarized the pharmacological effects and molecular mechanisms underlying of the plant-derived bioactive component SCL for further investigating its role in cancer and other diseases.

## Pharmacological activities of sclareol

### Anti-cancer effects

**FIGURE 1:**
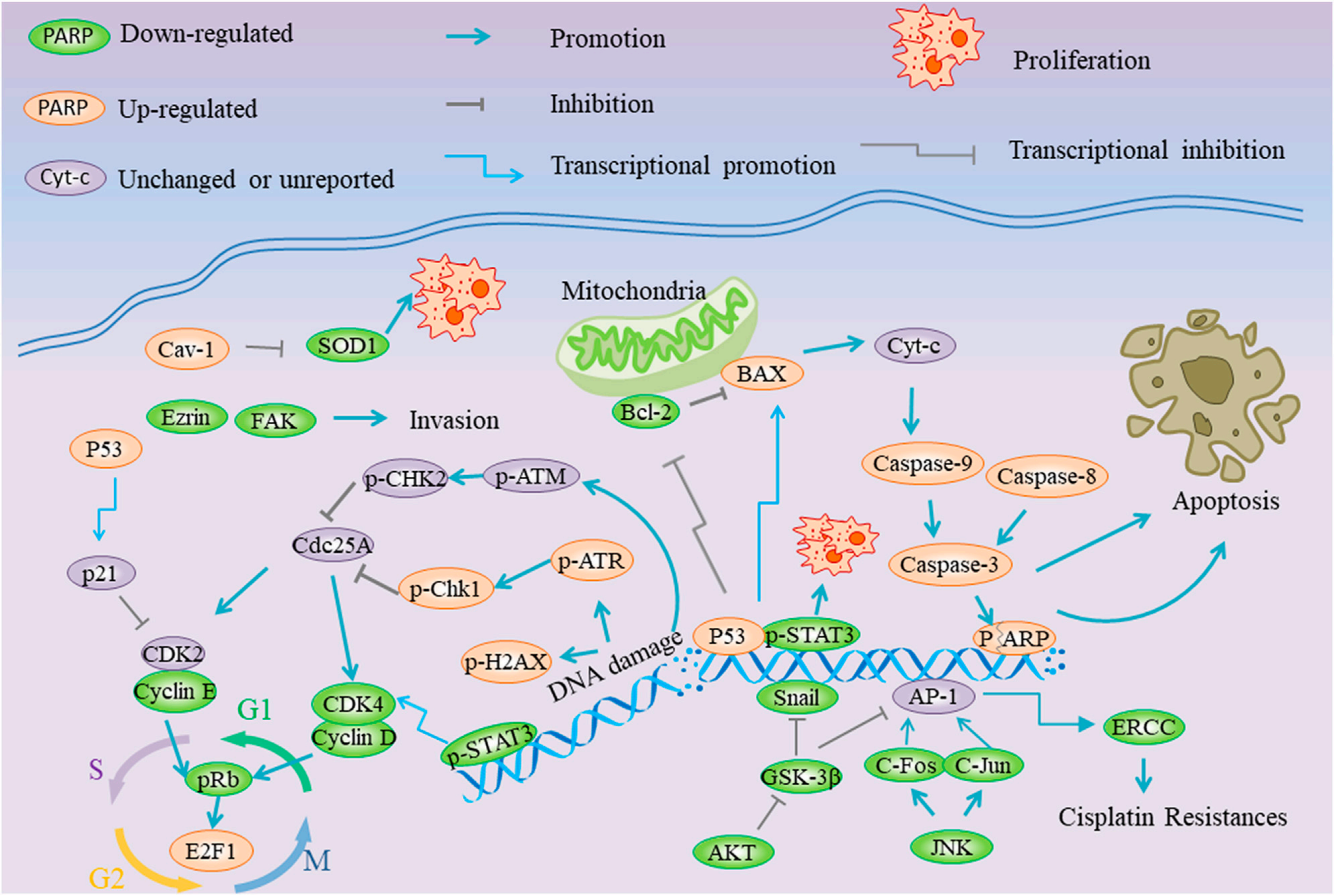
Schematic overview of the effects and molecular mechanisms of Sclareol in cancers.

As shown in Figure 1 and Table 1, SCL has performed extensive activities against cancer *via* multiple signaling pathways involving cell proliferation, apoptosis, cell cycle arrest and so on. The SCL performed proliferation-suppressive effects in various cancer cells (50% of inhibitory concentration, IC_50_ < 50 µm), including lung cancer, colon cancer, breast cancer (Paradissis et al., 2007). In addition, cell viability assay showed that splenocytes obviously ascended after SCL treatment while cell proliferation of K562 was restricted (Noori et al., 2013).

**TABLE 1:** The effects and mechanisms of SCL against various cancers *in vivo* and *in vitro*.

Cancer Type	Model	Dose	Effects	Mechanisms	Ref
Leukemia	HL60	10 μg/ml	Proliferation ↓; G0/G1 cycle arrest and DNA cleavage ↑	No reported	Dimas et al. (1999)
Breast	MN1, MDD2	50,100 µm	DNA synthesis inhibition, cell cycle arrest in G0/G1 phase, Apoptosis ↑	No reported	Sashidhara et al. (2007)
	MCF-7	30 μm (IC_50_: 31.11 μm)	Proliferation↓; Apoptosis↑	Bcl-2, p-STAT3 ↓; P53, BAX, Caspase-8, Caspase-9 ↑	Afshari et al. (2020)
Colon	HCT116	100 µm	G1 phase cycle arrest, DNA damage, Apoptosis ↑	Caspase-3, 8, 9 ↓; cleaved PARP ↑	Dimas et al. (2007)
Cervical	HeLa	5–20 μg/ml	Proliferation, chemosensitivity ↑	Cav1↑; SOD1 ↓; Cav1 downregulated SOD1 with lysosome-mediated ↑	Zhang et al. (2017)
Osteosarcoma	MG63	2, 5, 10 µm	Proliferation, Invasion ↓; Apoptosis ↑	Ezrin, FAK ↓	Mo et al. (2016)
	MG63	50,70,100 µm	mitochondrial membrane potential ↓; Apoptosis, G1 phase cycle arrest ↑	No reported	Wang et al. (2015)
Lung	H1688	25, 50,100 mm	G1 phase cycle arrest, Apoptosis, DNA damage ↑	CDK4, Cyclin D, Cyclin E, pRb, cleaved PARP, p-H2AX, p-ATR and p-Chk1↑; E2F1↓	Chen et al. (2020a)
	Cisplatin resistant A549	50, 100 μm	ERCC1↓; Drug sensitivity↑	GSK3β-AP1/Snail,JNK-AP1 ↓	Pan et al. (2020)
	H1688 mice xenograft model	300 mg/kg	tumor growth ↓	No reported	Chen et al. (2020a)
Breast	Spontaneous mouse mammary tumor	7.85 µg/mouse/day	IL-4,Treg↓; IFN-γ↑	No reported	Noori et al. (2010)
Colon	HCT116 bearing tumor mice	50 mg/kg	tumor growth ↓	Ki-67↓	Dimas et al. (2007)

Annotation: ↓, downregulated; ↑, upregulated; ref. reference.

Early studies suggested that SCL had anti-proliferation activity on leukemia cells (IC_50_ below 20 μg/ml at 48 h), induced G0/G1 cycle arrest and DNA cleavage in HL60 cells (Dimas et al., 1999). In breast cancer cell lines MN1 and MDD2, SCL (50,100 µm) triggered the DNA synthesis inhibition, cell cycle arrest in G0/G1 phase and cell apoptosis. Docking investigations *in silicon* revealed SCL putatively targeted BRCA1 with high binding affinity in natural compounds (Hossain et al., 2022). Besides, the 13-epimer-sclareol exerted antiproliferative effect against MCF-7 cells (IC_50_ = 11.056 μm) and induced apoptosis (10, 20 μm) (Sashidhara et al., 2007). Cellular study found that SCL induced G1 phase cycle arrest, DNA damage, and led to apoptosis by activating Caspase-3, 8, 9 and cleaved PARP in colon cancer HCT116 cells (100 μm) (Dimas et al., 2007).

The Caveolin-1(Cav1) and Superoxide Dismutase 1(SOD1) were supposed to as potential tumor suppressor and oncogene respectively. In cervical cancer cells, SCL (5–20 μg/ml) induced proliferative inhibition *via* promoting Cav1 expression and down-regulating SOD1, enhanced sensibility of MCF-7, HepG2, SW480 and SW620 cells to bortezomib. Interestingly, Cav1 was negatively associated with SOD1 through involving the lysosome-mediated degradation of SOD1, the effect was facilitated by SCL(Zhang et al., 2017). Additionally, SCL inhibited proliferation (IC_50_ = 14 μm), invasion and induced apoptosis (2, 5, 10 µm) in MG63 osteosarcoma cells, with the expression of Ezrin and FAK suppressed (Mo et al., 2016). Another similar study in MG63 cells implicated that SCL performed antiproliferative effect (IC_50_ = 65.2 µm) and induced apoptosis, G1-phase cell cycle arrest and loss of mitochondrial membrane potential (Wang et al., 2015).

Moreover, the synergistic effect of SCL (50 µm) and cisplatin, doxorubicin and etoposide ameliorated drug sensitivity of breast cancer (Dimas et al., 2006). Furthermore, the up-regulation of P53, BAX, Caspase-8, Caspase-9 and down-regulation of Bcl-2 was perceived to trigger apoptosis in breast cancer MCF-7 under SCL treatment, while SCL inhibited proliferation (IC_50_ = 27.65 μm) by suppressing the phosphorylation of STAT3, which enhanced by the combination of SCL and cyclophosphamide in the above regulative effect (Afshari et al., 2020). The cisplatin (6 mg/kg) combined with SCL (200 mg/kg) exhibited stronger tumor toxicity than cisplatin or SCL alone in A549 mice model with the down-regulation expression of cisplatin-resistant maker ERCC1. And the combination of SCL (100 μm) and cisplatin (50 μm) showed synergetic effect against survival and invasion of A549 cells. In mechanism, SCL (50, 100 μm) inhibited ERCC1 protein expression to sensitize A549 towards cisplatin treatment through attenuating ERCC1 upstream GSK3β-AP1/Snail and JNK-AP1 axis (Pan et al., 2020).

*In vivo*, SCL repressed the tumor growth by decreasing IL-4 and increasing IFN-γ level in breast cancer mice model, and notably suppressed the population of T regulatory cells (Treg) in tumor (Noori et al., 2010). SCL restricted tumor growth in xenograft model of small cell lung cancer H1688 cells, inhibited proliferation of H1688 cells and H146 cells with IC_50_ of 42.14 and 69.96 μm at 24 h respectively. In addition, SCL induced G1 phase cycle arrest with the decreased level of CDK4, Cyclin D, Cyclin E, pRb and the increased level of E2F1. Apoptosis that SCL trigging also been reported with caspase-3 activity promoted and cleaved PARP expression elevated, and SCL elevated p-H2AX, p-ATR and p-Chk1 expression to trigger DNA damage in H1688 cells (25, 50, 100 µM) (Chen H. L. et al., 2020).

### Anti-inflammatory effects

The anti-inflammatory effects of Labdane diterpenes through regulating NF- κB, nitric oxide (NO) and arachidonic acid metabolite axis had been reported, comprising andrographolide, andalusol, etc (Tran et al., 2017). As a part of labdane diterpenes family, SCL (intraperitoneal injection, 50 and 100 mg/kg) significantly attenuated inflammatory severity by inhibiting NF-κB translocation and phosphorylation of MAPK signaling in atopic dermatitis -like skin lesions model mice induced by 2,4-dinitrochlorobenzene, with local pro-inflammatory cytokine concentration reduced and T cell activation and cytokine production (IFN-g, IL-4 and IL-17 A) inhibited (Wu et al., 2019). SCL suppressed LPS-induced lung injury in mice *via* impeding NF-κB, MAPKs and HO-1 signaling transductions (Hsieh et al., 2017).

Additionally, SCL treatment retarded arthritic severities in mice model of rheumatoid arthritis through regulating inflammatory cytokines and the population of Th17 and Th1 cells. *In vitro*, SCL weakened IL-1β-induced expression of MMP-1, TNF-α, and IL-6 in SW982 cells *via* attenuating translocation of NF-κB and p38 MAPK/ERK/JNK pathways (Tsai et al., 2018).

Distinguishingly, the SCL induced eryptosis with the dysfunction of membrane phosphatidylserine in human erythrocytes, and partially regulated p38 kinase and casein kinase 1α (Signoretto et al., 2016). Interestingly, SCL inhibited RANKL-induced osteoclastogenesis and osteoclast function *in vitro* (1–10 μm), which was associated with SCL-triggering the suppression of NF-κB and MAPK/ERK signaling pathways, and prevented ovariectomy -induced mouse model from bone loss *in vivo* (Jin et al., 2019). SCL has been reported to improve dysmenorrhea and inflammation in dysmenorrhea models *in vitro* and *in vivo via* suppressing the Ca2+/MLCK/MLC20 pathway cascades (Wong et al., 2020).

The SCL performed anti-osteoarthritic activities by up-regulating TIMPs and inhibiting iNOS, COX-2 and MMPs expression in interleukin-1β-induced rabbit chondrocytes and knee osteoarthritis model of rabbit (Zhong et al., 2015). Moreover, sclareol (10 mg/kg) was found to ameliorate LPS-induced lung injury in mice through the suppression of NF-κB and MAPK signaling and activation of heme oxygenase-1 (HO-1) expression (Hsieh et al., 2017). In addition, the mechanism research indicated that the anti-inflammatory bioactivity of SCL was contributed to inhibition of inflammatory cytokines and enhancement of antioxidant enzyme activity. Sclareol inhibited the release of NO, TNF-α and MDA in the carrageenan-induced paw edema model, and restricted the cell growth and the expression of NO, iNOS and COX-2 in LPS-stimulated RAW264.7 macrophages (Huang et al., 2012).

### Anti-pathogenic microbes

The anti-microbial effect of SCL against *Candida* yeasts, including *C. albicans*, *C. glabrata*, *C. parapsilosis*, and *C. tropicalis* was almost equivalent to Fluconazole (Popova et al., 2019). The structure-activity relationship study found that the modification of branched chain and benzene ring in SCL improved its antifungal activity (Ma et al., 2018). Miaofeng et al. reported 20 derivates of SCL, in which compound 16 performed the best fungicidal activity against *Curvularia lunata* (IC_50_ = 12.09 μg/ml) and *Alternaria brassicae* (IC_50_ = 14.47 μg/ml) comparing with SCL and fungicide thiabendazole (Ma et al., 2015). Moreover, the SCL was first reported to inhibit helminth growth in larval (IC_50_ ≈ 13 μm), juvenile (IC_50_ = 5.0 μm), and adult (IC_50_ = 19.3 μm) stages of *Schistosoma mansoni*, a pathogen of schistosomiasis. Among 14 derivates of SCL, the most effective compound 12 enhanced cytoxicity against larval (IC_50_ ≈ 2.2 μm), juvenile (IC_50_ = 1.7 μm), and adult schistosomes (IC_50_ = 9.4 μm) by interfering with arachidonic acid metabolism to regulate membrane lipid homeostasis (Crusco et al., 2019). Importantly, the wide-spectrum effect against filoviruses of SCL has been proposed, especially, SCL was considered as Ebola virus (EBOV) entry inhibitor by interfering the viral fusion process (EC_50_ = 2.4 μm) (Chen Q. et al., 2020). In antibiotic resistance, SCL performed synergistic effect with clindamycin against Methicillin-resistant *Staphylococcus aureus* (Iobbi et al., 2021)*.* SCL also exerted antifungal synergies with Curcumin towards various fungus, including *Candida albicans*, *C. glabrata*, *Aspergillus fumigatus* (Augostine and Avery, 2022). The derivates of SCL were reported more effective against plant pathogenic fungal *A. alternate* and *A. brassicae* than thiabendazole (Ma et al., 2018).

### Anti-hypertensive and anti-diabetic effects

The reduction of blood pressure SCL induced was observed in normotensive and hypertensive rats, the phenomenon was probably due to ameliorated vasodilation *via* NO/cGMP signaling (Campos et al., 2017). The regulation of blood pressure mediated by SCL indicates it may be applied to cardiovascular disease as potential hypotensor. In addition, SCL was viewed as one of the bioactive components in *Salvia miltiorrhiza* and *Dalbergia odorifera* against miocardial infarction (Zhao et al., 2022). SCL improved hyperglycemia-induced renal injury (renal dysfunction, fibrosis, and inflammation) to prevent diabetic nephropathy through inducing inactivation of MAPKs and NF-κB pathway (Han et al., 2022).

## Pharmacokinetics, derivatives and pharmaceutical

Pharmacokinetic studies suggested that SCL was mainly distributed in extracellular fluid (apparent distribution volume was 21.4 L/kg), and its half-life was short (6.0 h) in rats (intravenous injection, 5.0 mg/kg) (Xiang et al., 2021). The neurotoxicity of free SCL was found in bearing tumor mice of colon cancer HCT116 cells when over 560 mg/kg, whereas 50 mg/kg SCL observed to be ineffective in toxicity (Paradissis et al., 2007). The low bioavailability attributed to its poor water solubility (0.0012 g/L) was considered as the main obstacle limiting its clinical application. The structure modification and nano-delivery systems were imported for enhancing bioactivities and pharmacokinetic properties, such water-solubility and distribution.

The aryl derivatives of SCL were synthesized by Heck coupling reaction for importing aryl in the end of SCL branch chain, in which the compound 15-(4-fluorophenyl)-sclareol (SS-12) exhibited the most effective anti-proliferation activity against PC3 cells (IC_50_ = 0.082 μm). SS-12 (0.3 μM) reshaped the balance between autophagy and apoptosis by regulating the BH3 domain protein Bcl-2 and Beclin 1. SS-12 (0.1–0.3 μm) induced autophagic cell death with the decreased level of P62 and increased expression of LC3-I, LC3-II, Beclin-1, while triggered apoptosis by blocking the Akt/mTOR pathway in PC-3 Cells (Shakeel u et al., 2015). The tumor growth of Sarcoma-180 Solid and Ascitic Tumors was dramatic suppressed on the group of SS-12 (5, 10 mg/kg i. p.) comparing with the control group treated with 5-fluorouracil (22 mg/kg i. p.) or normal saline.

Highly lipophilic sclareol was encapsulated in PLGA nanoparticles, and then the surface of nanoparticles was modified by hyaluronic acid (HA) to construct HA-NanoSCL for targeting hyaluronic acid receptor in breast cancer. The HA-NanoSCL nanosystem enhanced cytotoxicity against MCF-7 and MDA-MB-468 (0–50 µm) and uptake of SCL in MDA-MB-231 cells (Cosco et al., 2019). Interestingly, The natural and environmental-friendly nano-formulation was reported that SCLAREIN (SCL encapsulated by plant protein zein) with mean size of 120 nm, performed great stability and time-dependent release in 1 week, while the nanoparticles (loading 1 mg/ml SCL) possessed stronger cytoxicity of MCF-7 and K562 than free SCL (Gagliardi et al., 2021).

The liposome, lipid nanoparticles (LNPs) and nanostructured lipidic carriers (NLCs) have been viewed as carriers for lipophilic SCL delivery based on SCL low water solubility and high lipophilicity. Liposomes targeting mitochondria significantly improved the apoptosis induction and cytotoxicity of SCL (Patel et al., 2010). Moreover, liposome SCL increased the distribution of SCL in the nucleus of colon cancer HCT-116 cells (Paradissis et al., 2007) and reduced the tumor growth in HCT116 xenograft mice (Dimas et al., 2007). Solid lipid nanoparticles (SLN) loading with SCL exerted excellent physicochemical features including encapsulation efficiency (EE, 89%) and drug loading (DL, 42.47 mg/g), and realized sustained drug release over 1 week and time-dependent proliferative inhibition in A549 cells comparing with plain SCL (IC_50_ = 19 μg/ml) (Hamishehkar et al., 2018). Similarly, SLN encapsulated adriamycin and SCL enhanced the antitumor effect of doxorubicin compared with free adriamycin in breast cancer 4T1 cells (Oliveira et al., 2018).

To conquer the drug resistance in cancer and facilitate chemotherapy response, combination therapy has been widely used in clinical and basic investigation. SCL was reported as an enhancer of doxorubicin (DOX) and the combination of DOX and SCL showed stronger anti-proliferative effect than free DOX and free SCL in breast cancer MDA-MB-231 and 4T1 cells. In 4T1 mice model, the nanostructured lipid carrier loading Doxorubicin and SCL (NLC-DOX-SC) exhibited better tumor inhibition than plain DOX and NCL-DOX, also performed lower cytoxicity than the combination of free DOX and SCL in weight loss and myelosuppression (Borges et al., 2019). However, recent research indicated that NLC-SCL exerted higher encapsulation than SLN-SCL, which was contributed to the difference of lipid matrix (Borges et al., 2021). NLC-SCL performed higher anti-proliferation effect than plain SCL against MDA-MB-231 and HCT-116 cells. Moreover, NLC-SCL G2/M phase arrest in above cells (Borges et al., 2021). Variously, sclareol-loaded lipid nanoparticles effectively improved metabolism and attenuated obesity process in obesity induced mice, which was attributed to the decreased expression of proinflammatory cytokines (NF-kB and MCP-1) and adipogenesis related markers SREBP-1 (Cerri et al., 2019).

## Conclusion and prospect

Although sclareol has exhibited extensive and wide-spectrum effects for attenuating cancer-related phenotypes, such as proliferation, apoptosis and cell cycle, the molecular pathways sclareol mediated remain uncharted and the present studies focusing on signaling mechanisms are not deep and comprehensive. For instance, weather SCL is associated with ferroptosis and pyroptosis, and the relationship between SCL with m6A RNA methylation remains underlying. Investigations on structure activity relationships provide new sight to uncover the bioactivities between SCL and its analogues, while the improvement of pharmacokinetic parameters (water solubility and half-life) and targeting are entitled by delivery systems, including liposome, lipid nanoparticle, etc. Beyond anti-tumor effects, SCL also exhibited other attributes, mainly comprising anti-inflammation and anti-pathogenic microbes (fungal, schistosomiasis and Ebola virus). The classic NF-κB and MAPK signaling pathways exerts crucial role in the anti-inflammation property of SCL. It is significant that SCL triggered immunomodulatory effects of Th17, Th1 and Treg may involve tumor microenvironment remodeling. Promisingly, SCL was identified as a novel Cav1.3 antagonist against Parkinson’s disease (Wang et al., 2022). SCL was considered as candidate drug to treat or prevent SARS-CoV-2 *via* targeting Covid19 Main Protase (MPro) (Aydın et al., 2021). Sclareol as F1Fo-ATP synthase inhibitor restrained free radical production in the retinal rod, which indicated SCL could serve as a potential drug for retinal disease (Ravera et al., 2020).

Biosynthetic strategy provided new application prospect for industrial manufacture of sclareol with green and sustainable, compared to traditional extract from plants (Einhaus et al., 2022). We look forward emerging investigations to further explore the role of sclareol in combined therapy with chemotherapy or immunotherapy against cancer, even in Covid19 and Parkinson’s disease.
